# *In silico* investigation of molecular networks linking gastrointestinal diseases, malnutrition, and sarcopenia

**DOI:** 10.3389/fnut.2022.989453

**Published:** 2022-11-04

**Authors:** Matti Hoch, Luise Ehlers, Karen Bannert, Christina Stanke, David Brauer, Vanessa Caton, Georg Lamprecht, Olaf Wolkenhauer, Robert Jaster, Markus Wolfien

**Affiliations:** ^1^Department of Systems Biology and Bioinformatics, University of Rostock, Rostock, Germany; ^2^Division of Gastroenterology, Department of Medicine II, Rostock University Medical Center, Rostock, Germany; ^3^Leibniz-Institute for Food Systems Biology at the Technical University of Munich, Freising, Germany; ^4^Wallenberg Research Centre, Stellenbosch Institute for Advanced Study, Stellenbosch University, Stellenbosch, South Africa; ^5^Faculty of Medicine Carl Gustav Carus, Institute for Medical Informatics and Biometry, Technical University Dresden, Dresden, Germany

**Keywords:** sarcopenia, malnutrition, gastrointestinal diseases, systems biology, network modeling

## Abstract

Malnutrition (MN) is a common primary or secondary complication in gastrointestinal diseases. The patient’s nutritional status also influences muscle mass and function, which can be impaired up to the degree of sarcopenia. The molecular interactions in diseases leading to sarcopenia are complex and multifaceted, affecting muscle physiology, the intestine (nutrition), and the liver at different levels. Although extensive knowledge of individual molecular factors is available, their regulatory interplay is not yet fully understood. A comprehensive overall picture of pathological mechanisms and resulting phenotypes is lacking. *In silico* approaches that convert existing knowledge into computationally readable formats can help unravel mechanisms, underlying such complex molecular processes. From public literature, we manually compiled experimental evidence for molecular interactions involved in the development of sarcopenia into a knowledge base, referred to as the Sarcopenia Map. We integrated two diseases, namely liver cirrhosis (LC), and intestinal dysfunction, by considering their effects on nutrition and blood secretome. We demonstrate the performance of our model by successfully simulating the impact of changing dietary frequency, glycogen storage capacity, and disease severity on the carbohydrate and muscle systems. We present the Sarcopenia Map as a publicly available, open-source, and interactive online resource, that links gastrointestinal diseases, MN, and sarcopenia. The map provides tools that allow users to explore the information on the map and perform *in silico* simulations.

## Introduction

Malnutrition (MN) is a common and characteristic feature of gastrointestinal diseases, such as liver cirrhosis (LC) and intestinal dysfunctions (ID), e.g., short bowel syndrome (SBS), and is associated with high mortality rates (1). For LC patients, the prevalence of MN is indicated with up to 90% (2); for patients suffering from SBS with around 10–40% (3). Disease-related MN is closely related to mild, chronic inflammation (4). Both MN and inflammation contribute to muscle wasting, which, combined with the loss of muscle function, can eventually result in sarcopenia. This vicious cycle of MN, inflammation, sarcopenia, and the underlying disease itself leads to an unfavorable prognosis for the patient (5). A sufficient supply of energy and nutrients is needed for homeostasis of muscle anabolism and catabolism. Conversely, an inadequate nutrient uptake by intestinal malabsorption and a deficient metabolism of nutrients, as well as deficient breakdown of muscle waste products in the liver can impair muscle growth (6, 7). Additionally, microbial invasion caused by a disrupted epithelial barrier in ID and LC leads to systemic inflammation that stimulates catabolic processes in the muscle (5, 8, 9). The liver, as a main producer of cytokines and hormones, also releases many pro-inflammatory mediators during injury that favor muscle atrophy (7, 10, 11). The fact that the control of muscle physiology is consequently highly dependent on intestinal and liver function, makes sarcopenia a common secondary phenomenon in ID and LC (5).

Given the physiological and pathophysiological association of intestine, liver, and muscle function, it is not surprising that they are linked by complex molecular communication networks (12–14). Although the role of many molecules has been elucidated by extensive *in vitro* and *in vivo* experiments, understanding the system as a whole, including all the interactions involved, is a task beyond human capabilities. Therefore, the use of *in silico* approaches, i.e., the conversion of available knowledge into computationally readable formats, can help unravel this complexity. In this context, Systems Biology models have already been used to study complex systems, such as nutrient absorption (15), muscle fiber physiology (16, 17), pathologic liver metabolism (18), and diabetes (19). Although these models enable detailed simulations by integrating kinetic information, they are inherently limited to small-scale applications, such as spatially defined signaling processes. To this end, a resource that links gastrointestinal diseases, nutrition, and muscle (patho-)physiology on a larger scale and enables simulations across tissues is lacking.

**Disease maps** have emerged as web-based resources collecting information on molecular interactions to enable disease-specific interactive visualizations and computer-based simulations (20). Prominent examples of established disease maps include the Atlas of Inflammation Resolution (AIR) (21), the Parkinson’s Disease Map (22), the Rheumatoid Arthritis Map (23), the AsthmaMap (24), the Atherosclerosis Map (25), or the COVID-19 disease map (26). Many of those have been published on MINERVA, a web platform that allows to develop disease-specific analysis tools, making it an excellent framework for interactive visualizations of disease maps (27). The use of computational standards in MINERVA, e.g., the systems biology markup language (SBML), which describes how biological models are represented graphically and stored computationally, ensures reproducibility (28, 29). Through cell type-, tissue-, or process-specific modularization, thus creating so-called submaps, disease maps help to provide an intuitive overview of complex disease mechanisms. All submaps together form a single large-scale molecular interaction map (MIM) (21), which is directed graph encompassing all interactions that connect elements in the graph which represent biological entities such as proteins, small molecules, pathways, or diseases. Given the high number of interactions and scarcity of available data, it is extremely challenging to parameterize all interactions in the disease map. Therefore, approximations of non-parametric mechanisms in and in between submaps are required.

Topological analyses determine the interconnectedness of nodes in the network by traversing along the causal interactions (30, 31). In this way, relationships between distant elements can be detected, nodes in signaling pathways can be identified, or weights can be assigned to elements that regulate a particular process (32). Topological methods have also been used to extract core regulatory networks from large-scale networks to investigate mechanisms on a smaller scale (33). In addition, topological information have been used to improve the analytical performance of statistical enrichment (34) or machine learning approaches (35). Topological analysis is less complex but can be problematic in highly interconnected networks. Identifying all paths in larger networks, i.e., all connections between every pair of elements, is a computationally intensive challenge. Consequently, many algorithms focus on identifying only the shortest paths between two nodes in the network (36). Thus, topological analysis can be highly affected by biases such as (i) misestimating the length of interactions that lack intermediates, (ii) neglecting the biochemical relevance of longer pathways, and (iii) overrepresenting more intensively studied molecules. Nevertheless, they provide means for implementation and sufficient informative power to compare elements that are included in a given pathway and to what extent.

**Boolean models** are much better suited to study network mechanisms and to investigate the effects of molecular perturbations on the system (37, 38). In Boolean models, the **state** of each gene/molecule/phenotype is constrained to be either active (**ON/**1) or inactive (**OFF**/0), defined by specific Boolean rules based on the state of other network elements (39). In successive steps representing a time scale, the state of each element in the map is evaluated based on the states of incoming elements in the previous step. Since there is a finite number of possible network states, at some point a **steady state** is reached that is either stable, i.e., remains in one state, or oscillates, i.e., changes infinitely between one or multiple states. The steady state provides useful qualitative information on molecular mechanisms, in particular on circulating regulatory feedback and feedforward loops. Analysis of the number of active states during the steady state as a function of a given input makes it possible to determine correlations between elements regardless of their distance in the network (40, 41). This is of great importance in complex processes such as energy metabolism, where the influence of each nutrient must be considered equally at each time point. Moreover, in Boolean models, the computational time increases only proportionally to the complexity of the network, allowing efficient high-throughput analyses. The development of a Boolean model simulating the influence of nutrition and metabolism on sarcopenia may therefore prove useful in assessing the effects of various physiological and pathological conditions.

We developed an in-depth, standardized, and computationally encoded disease map of the molecular environment that regulates sarcopenia, which we term the “Sarcopenia Map,” and integrated the two disease states ID and LC. Given their relevance in the development of sarcopenia, we modularized the map into three tissue-specific submaps for (i) the **intestine**, i.e., nutrient uptake, its hormonal regulation, and the effects of ID, (ii) the **liver**, i.e., metabolic processes, cytokine secretion, and their alteration in LC, (iii) and the **muscle**, i.e., molecular regulation of catabolic and anabolic muscular processes leading to sarcopenia. In addition, we converted the underlying interaction network into a Boolean model and validated the model by simulating clinically relevant molecular perturbations. We integrated our methods into an interactive MINERVA tool suite allowing researchers to explore the information on the maps, identify interaction pathways, and perform *in silico* perturbation experiments. With these tools, we demonstrate how the map contributes to understanding the complex molecular processes leading to sarcopenia.

## Materials and methods

### Map curation

**FIGURE 1:**
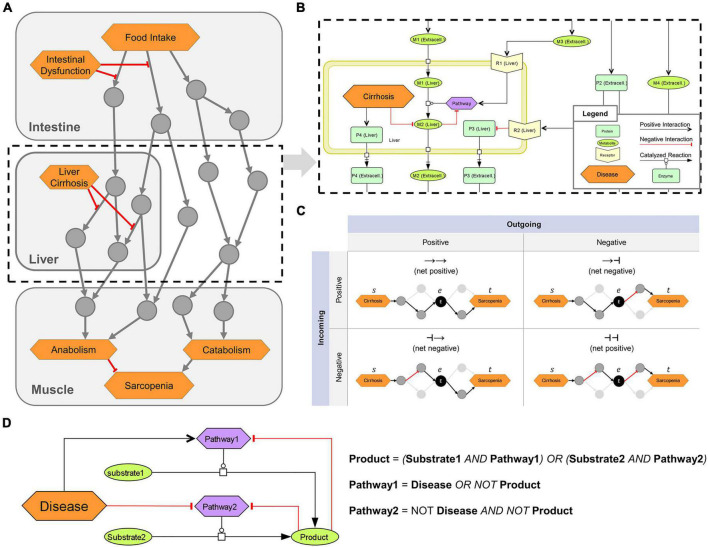
Overview of the logical modeling approaches in the sarcopenia map. **(A)** Schematic structure of the Sarcopenia Map. **(B)** SBML schema of tissue-specific compartments (yellow frames) that are connected through extracellular elements, e.g., representing hormones or cytokines. **(C)** Topological analysis of the underlying network. Paths between two elements are analyzed based on their length, type (positive or negative), and passed nodes. Elements are ranked by their inclusions in the identified paths. **(D)** Creation of Boolean rules that define an element’s state by converting SBML reactions into logical gates.

We screened the PubMed database for published literature focusing on recent reviews describing the intestinal uptake of nutrients and their metabolism in the liver, hormonal communication between liver and muscle, and regulation of muscle growth and function. Simultaneously, we sought information on the effects of ID and LC on these processes. The information was then further examined to ensure that the interactions identified were direct, such as protein-receptor interactions. To improve clarity and ease curation efforts, we collected the information in three Systems Biology Markup Language (SBML)-standardized **submaps** in CellDesigner (29, 42). Intracellular molecules were enclosed in compartments reflecting the organ, while extracellular molecules were placed outside the compartments, either representing molecules in the bloodstream (e.g., nutrients or cytokines) or systemic conditions, such as acidosis or hyperammonemia. This separation enables the distinction between tissue-specific processes and connects them through the intervening communication processes. Figure 1A provides a schematic overview of the map organization and the hierarchical flow of information through the submaps.

Different shapes and colors enable intuitive visualizations of various biological or clinical entities, including (i) molecules, such as genes, proteins, or metabolites; (ii) their subclasses, such as receptors and ion channels; (iii) clinical features; and (iv) whole pathways. We refer to all of these entities collectively as **map elements** (Figure 1B). We connected the elements by SBML-standardized reactions representing their biochemical interaction. We simplified most reactions in the activity flow format, i.e., represented more complex mechanisms (e.g., phosphorylations) as single arrows connecting a source element (e.g., the protein kinase) with a target element (e.g., the protein). This simplification reduces the map content and improves readability while retaining all necessary information. Only enzymatic reactions were retained in the process description format because information about enzymes is necessary for modeling the mechanisms of metabolic regulations. Larger metabolic pathways, e.g., glycolysis, have been combined into a single catalytic reaction leading from the initial reactant (glucose) to the final product (pyruvate), omitting all intermediates. The reaction is catalyzed by a phenotypic element (glycolysis) that represents the metabolic pathway *per se*. All regulations, e.g., product-feedback-inhibitions or hormonal, were then added as reactions to the phenotype element (exemplarily shown in Figure 1B).

### Network generation

We transformed the submaps into a single graph (G) consisting of a set of elements [vertices V(G)] connected by interactions [edges E(G)]. All reactions in the submaps were converted into one or multiple interactions each consisting of two elements that are linked by either upregulation (positive) or downregulation (negative). Therefore, *E*(*G*) is defined as a collection of triples *E* ⊂ (*s*|*r*|*t*) consisting of a source element *s* ∈ *V*, a relation *r* ∈ {−1, 1} representing a positive (activating) or negative (inactivating) interaction, and a target element *t* ∈ *V*. All enzymatic reactions in the maps, which catalyze the synthesis of a product *p* from a substrate *s* by an enzyme *e*, were transformed into an edge triplet of (s|1|*p*), (e|1|p), and (e|−1|s). The latter represents the consumption of the substrate by the enzyme. For reactions with multiple substrates, all substrates were first combined into a complex with edges connecting the substrates and the complex. The complex *c* then acts as the substrate of the reaction triplet. For reactions with multiple products, reaction triplets were generated for each product. A path *P*(*G*) of the length *L* ∈ ℕ can be written as the sequence (*u*_1_ ⟶ ^*r*_1_^ *u*_2_ ⟶ ^*r*_2_^ ⟶ ^*r_L_*^ *u*_*L*+1_) with (*u*_*i*_, *r*_*i*_, *u*_*i*+1_) ∈ *E*. The type *T* ∈ {−1, 1} of any *P* is defined as (*r*_1_ ⋅ *r*_2_ ⋅…⋅ *r*_*L*_).

### Topological modeling

We identified paths in the MIM using a breadth-first-search (BFS) algorithm, one of the fastest possible solutions in a directed and unweighted graph (43). In its standard form, the algorithm enables the search for shortest paths (*SP*) between two elements (*u*, *v*) ∈ *V* as a set of existing paths *P*_*u,v*_ between *u* and *v*, where *L*(*P*_*u*,*v*_) is minimized. To identify more paths between *u* and *v*, we adapted the BFS algorithm to stop at already visited interactions instead of visited elements. The set of all identified paths or SPs that connect at least two specified elements, we call a pathway in the graph. To determine the role of an element *e* in the pathway of *u* and *v*, we filtered the paths between *u* and *v* by those that go through *e*. In addition, the filtered paths were split into two subpaths, *P*_*s,e*_, from *s* to *e* (incoming), and *P*_*e,t*_, from *e* to *t* (outgoing), with *T*(*P*_*s*,*t*_) = *T*(*P*_*s*,*e*_) ⋅ *T*(*P*_*e*,*t*_) and *L*(*P*_*s*,*t*_) = *L*(*P*_*s*,*e*_) + *L*(*P*_*e*,*t*_) (Figure 1C). This separation of paths provides us with information on (i) the ratio of positive and negative paths between *s* and *t*, in which *e* is involved, and (ii) the ratio of how *e* is regulated by *s* and how *e* regulates *t*. Repeating this analysis for other elements in the MIM compares their role in the investigated pathway.

### Boolean modeling

Based on the interactions in the submaps, we defined a Boolean rule for each element that specifies how its state (either ON or OFF) is defined by the state of other elements (inputs) represented by logical gates (NOT, OR, or AND) (Figure 1D). A Boolean rule may consist of multiple gates, which may be nested. When a reaction requires multiple elements to be active, such as in enzymatic reactions or the formation of complexes, these elements are represented by AND gates. Any negative input, such as from a disease or negative feedback, is integrated as a NOT gate. In general, all logic gates must be satisfied for an element to be ON, with disease inputs taking precedence. An exception, however, is the glycogen element in the model, whose state we represent as an integer that increases by 1 at each step at which the element’s Boolean rule is satisfied and decreases by 1 when it is not. As long as its state is greater than zero, it is treated as an ON input to other elements. In this way, we can simulate the construction of a storage and its subsequent use, even after its inputs subsided. Finally, we defined an initial state of the model, in which some elements with no inputs, such as digestive enzymes or transporters, are set to ON. Supplementary material shows the list of map elements with their initial state.

### Correlation analysis

We identified the correlations between two elements based on the dependency of their activity. The principle behind this methodology has been described by Helikar and Rogers (41). The activity of an element is defined as the percentage *p* of active states of the model during a range of *n* observed steps (*n* = 100 by default). We perturbed a source element *s* either through a set activity or through inhibition. Setting the activity of an element means changing its state to OFF and then to ON at every k-th step depending on the activity frequency *p*_*a*_(*s*) with $k=\frac{1}{p_{a}\,\left(s\right)}$. For example, the input activity frequency *p*_*a*_(*s*) = 0.25 refers to a state sequence for *s* of [1-0-0-0-1-0-0-0-1-…]. When perturbing *s* through inhibition, its state is set to OFF for every *k*-th step with *k*=$\frac{1}{p_{i}\,\left(s\right)}$, while in all other steps the element behaves normally. Then after performing *n* steps, we measured the activity of the target element *t* as the percentage of steps with ON state. If *t* is the “sarcopenia” phenotype in the muscle, its activity is defined as


p⁢(sarcopenia)=p⁢(catabolism)-p⁢(anabolism)

The phenotypes “anabolism” and “catabolism” themselves are defined as:


p⁢(catabolism)=p⁢(apoptosis)+p⁢(proteolysis)

and


p⁢(anabolism)=p⁢(cell⁢differentiation)+p⁢(protein⁢synthesis)

The perturbation simulation was then repeated for different activities of *s*. The correlation between both elements’ activities in each simulation was then analyzed using the “pearsonr” function from the stats module of the scipy python package generating the Pearson correlation coefficient and a two-sided *p*-value. Since Boolean models are susceptible to interferences, meaning that independent signals can overlap and distort the measurement, only a few observations could lead to incorrect assumptions. Thus, we use a wide range of activities for *s* (from 0 to 100% in 1% increments), which ensures that the observed correlations are more reliable.

## Results

**FIGURE 2:**
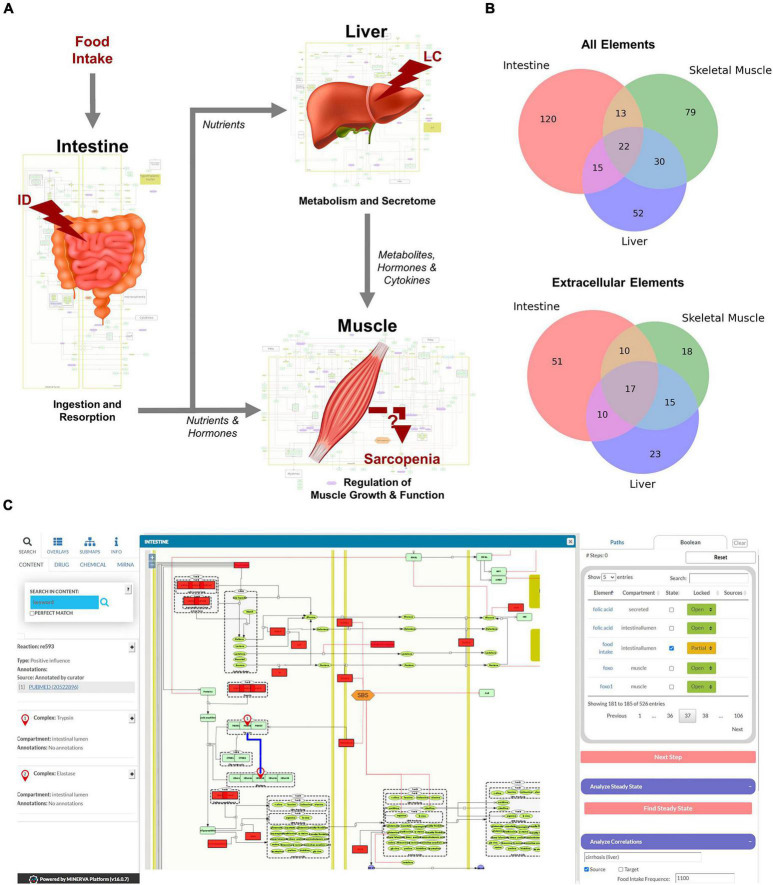
Overview of the hierarchical organization of the Sarcopenia map. **(A)** We summarized information on molecular interactions related to sarcopenia from the literature into three tissue-specific submaps. In addition, we integrated the effects of liver cirrhosis (LC) and intestinal dysfunction (ID) on these molecular processes. **(B)** Venn Diagrams of element distributions between the different tissues. **(C)** The interactive user interface of the Sarcopenia Map enables the exploration of information, as well as simulations of molecular perturbations.

We present the Sarcopenia Map^1^ as a publicly available, comprehensive knowledge base of experimental evidence for molecular interactions related to sarcopenia and linked to LC and ID (Figure 2). In the following, we explain in more detail (i) the Sarcopenia Map as a knowledge base (ii) its tools to perform *in silico* simulations, and (iii) applications and validations of the underlying computational model. We provide examples of how the tools help researchers to analyze disease mechanisms by investigating the molecular interactions linking nutrition, gastrointestinal diseases, and sarcopenia.

### A knowledge base of molecular interactions in sarcopenia

We have compiled findings from the scientific literature into three standardized, tissue-specific submaps (Figure 2A). The submaps summarize the processes in each tissue as SBML-standardized molecular networks (Supplementary Figures 1–3). Figure 2B shows the distribution of elements between submaps. Their overlap is more dominant for extracellular elements, which is to be expected since they represent secreted molecules that mediate communication between compartments. The highest number of tissue unique elements is found in the submap of the intestine, as it contains food components, as well as digestive enzymes and transporters.

MINERVA provides features to explore elements in the map and targets of specific drugs, miRNAs, or chemicals. The submaps are publicly accessible and can be downloaded in various formats (e.g., SBML, .svg, or .pdf). All elements and interactions in the maps are annotated with references to public databases or scientific literature (e.g., PubMed). The Sarcopenia Map comes with an interactive tool that we developed in this study to allow users to explore interaction paths in the sarcopenia map through topological analysis and to perform *in silico* simulations with Boolean models in an easy-to-use interface (Figure 2C).

### A platform for interactive *in silico* experiments

**FIGURE 3:**
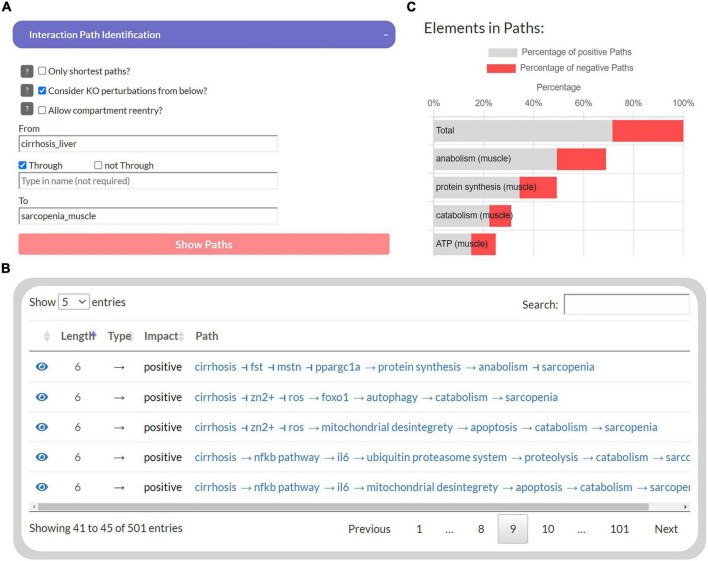
The user interface to identify interaction paths between selected elements in the Sarcopenia Map. For selected elements **(A)**, their interaction pathways are listed in a table **(B)** Additionally, elements along the pathways are ranked by their percentages of appearance separated by the type of interaction **(C)**.

The first part of the developed tool provides network topology functions to investigate interaction paths between user-specified elements to explore their underlying molecular regulations. Users can select a source element (“*From*”) and a target element (“*To*”) whose interaction paths are to be identified in the MIM (Figure 3A). In addition, another element can be specified to filter paths that pass “*Through*” that element. The output is presented as a table that shows all identified paths, their length, the total impact on the target, and all individual steps within the path (Figure 3B). Each interaction is referenced by a PubMed identifier and clicking on the icon takes the user to the location on the submaps. In addition, a bar chart lists the percentage of these paths, in which each element occurs, separated into positive and negative paths (Figure 3C). Because of the limitations of topological models (see “Introduction” section), assumptions about functional relationships and mechanisms should not be inferred from the distribution of positive and negative interaction paths alone. Nevertheless, they provide an intuitive overview of the design of molecular pathways and the flow of information.

**FIGURE 4:**
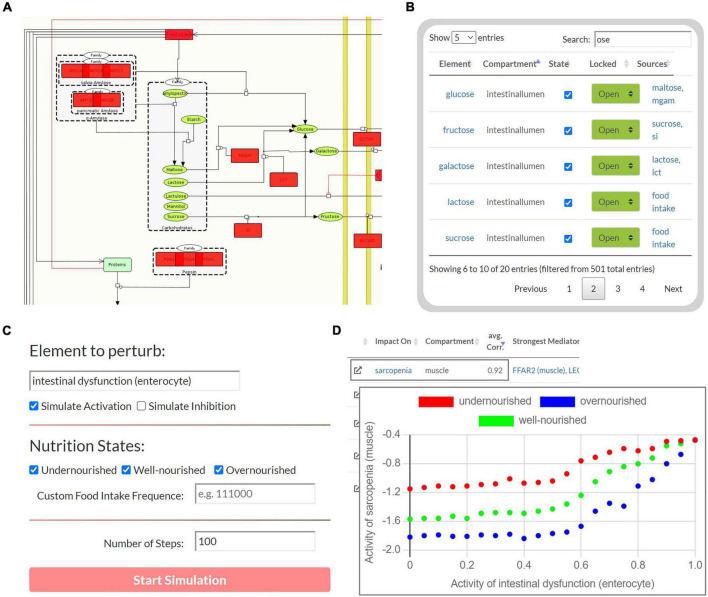
The user interface to perform Boolean simulations on the Sarcopenia Map. **(A)** For each step, the active (red) or disturbed (gray) elements in the network are highlighted. **(B)** An interactive table provides an overview of all elements in the maps and allows perturbations by activating or inhibiting their state. **(C)** Automated perturbation experiments allow simulation of an increase in activation or inhibition of a selected element. **(D)** The correlation of the activities of the other elements in response to the perturbation is then presented in a table and diagrams.

The second part of the tool enables Boolean simulations on the Sarcopenia Map *via* a simple user interface and colored overlays on the map (Figure 4). One of its functions is correlation analysis, which provides insights into the mechanistic relationship between elements. For a selected element (**source**) and nutrition states, multiple simulations are iteratively and automatically performed with increasing activity or deficiency of the source element (Figure 4C). At each iteration, the activity of other elements (**targets**) is measured during the resulting steady state. The correlations of the source and all target elements, represented by the Pearson correlation coefficient, are then summarized in a table. Scatter plots of the activities of the two elements provide further information by showing the detailed correlation course at different nutrition states (Figure 4D). For every target element, the table also ranks other elements in the network according to the similarity of their activity distributions toward the source and target. Elements that correlate with both, are most likely responsible for transmitting the signals. In addition, based on the type of correlation (positive or negative), we can investigate the role of the transmitting element, i.e., whether inhibition/activation of an inhibitory/activating signal has occurred or *vice versa*. Another function of the Boolean model is the identification of steady states. Supplementary Figure 4 illustrates the activity of elements during the oscillating steady state, which evolves from the default input state of the map by simulating a permanently active food input. The figure shows that most of the elements that change their state during the steady state are metabolites and metabolic enzymes. As long as food intake is constantly ON, extracellular glucose is as well, leading to a constant oscillation between glycolysis and glycogen synthesis. Feedback loops are essential for reversible responses in Boolean models, otherwise, the ON signal would be sent back and forth infinitely, even when the external signal is removed. Once we set the food intake to OFF and iterate forward, the new resulting steady state is stable, i.e., it does not oscillate and has no active metabolites (not shown).

### (Patho-)physiological simulations of nutrition and disease states

To test the Boolean model, we studied the behavior of the carbohydrate system under different nutrition states, i.e., different active frequencies of the “food intake” element. The carbohydrate metabolism is a tightly regulated system and the central part of the energy cycle that controls muscle function. Therefore, the carbohydrate system is a key pathway linking LC and ID to sarcopenia, as carbohydrate resorption, storage, and usage are impaired in these diseases (44, 45). In clinical settings, glucose supplementation has been shown to reduce muscle mass loss, while glycogen depletion has been identified as a major cause of the development of sarcopenia in LC patients (46, 47). We need to ensure that in our model carbohydrate activities respond correctly to changing nutritional conditions and perturbations.

**FIGURE 5:**
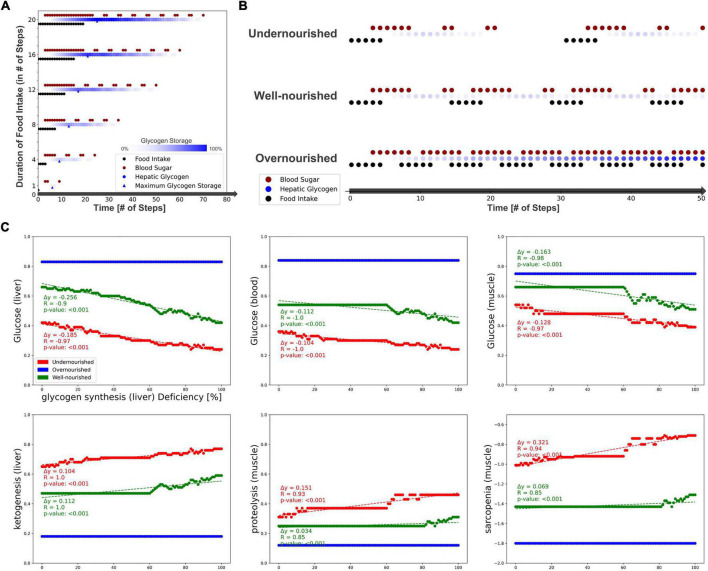
Testing the model by simulating different nutrition states and carbohydrate availability. **(A)** The activity of hepatic glycogen storage and extracellular glucose depending on the duration of the food intake stimulus. **(B)** Definition of three nutrition states by their food intake frequency and the resulting activities of hepatic glycogen storage and extracellular glucose. **(C)** Predicted activities of selected elements in response to an increasing deficiency of hepatic glycogen synthase.

First, we measured the response of glucose and glycogen to altered nutritional stimuli. Figure 5A shows the extent of hepatic glycogen storage (blue dots) and blood glucose (red dots) in response to increasing food intake (*y*-axis, black dots). As expected, we observed increasing hepatic glycogen activity and its prolonged conversion to blood glucose after food intake was switched off. Blood glucose is continuously active as long as food intake occurs and is oscillating during glycogen depletion. These results show that our model can simulate the conversion of glycogen to glucose and its release into the bloodstream in fasting situations. Next, we measured carbohydrate behavior again, but with different combinations of ON and OFF food intake, representing changing frequency and quantity, but not quality, of diet. From these, we identified three specific **nutrition states**, which will act as input for the model to simulate (patho-)physiological behavior. Importantly, Boolean models use steps as an discrete and arbitrary measurement of time and are not able to simulate real time-scale. Here, we define the nutrition states by their impact on the carbohydrate system (Figure 5B): (i) **undernourished**, i.e., long fasting periods with full depletion of glycogen storage (5 ON-steps and 25 OFF-steps), (ii) **well-nourished**, with continuous glycogen storage (5 ON-steps and 10 OFF-steps), and (iii) **overnourished**, with continuously increasing glycogen (5 ON-steps and 2 OFF-steps). We incorporated these states into the Sarcopenia Map user interface to facilitate their comparison when running different simulations. These nutritional states differ only in the quantity of food, not its composition, and are assumed to contain all macro- and micronutrients. However, users of the map can disable elements in the intestine submap to change the composition of the diet individually.

After testing the model under physiological conditions, we simulated pathophysiological disease states by molecular perturbations. We investigated how a deficiency of glycogen synthase (GS) in the liver correlates with the activity of glucose in the liver, blood, and muscle, ketogenesis in the liver, and proteolysis and sarcopenia in the muscle (Figure 5C). Most noticeably, in the overnourished state, GS deficiency has no correlations with any of the elements and, in the well-nourished state, a correlation becomes visible at high inactivation only. The latter is probably caused by the compensation of a lower GS deficiency by the increased blood glucose due to a more frequent food intake compared with the undernourished state. GS deficiency correlates negatively with glucose activity, which is most prominent in the liver compartment and less in the blood and muscle compartments. In the well-nourished state, glucose activity in the muscle shows a large plateau at medium GS deficiencies (20–60%), possibly due to compensation by muscle glycogen. A positive correlation is visible for the “ketogenesis” phenotype in the liver and “proteolysis” in the muscle, both physiological responses to hypoglycemic states (47, 48). Interestingly, the plot for “sarcopenia” also shows a positive correlation and is very similar to that for “proteolysis,” suggesting that sarcopenia in GS deficiency is most likely mediated by increased activity of muscle proteolysis. We conducted additional simulations for deficient glycogenolysis (Supplementary Figure 5A), deficient glucose uptake in the muscle (GLUT4/SLC2A4, Supplementary Figure 5B) and deficient glucose resorption in the intestine (SGLT1/SLC5A1, Supplementary Figure 5C). All three cases positively correlate with sarcopenia. Although both GLUT4 and SGLT1 deficiencies lead to glucose depletion in muscle, the effect of SGLT1 on sarcopenia is much stronger, especially in well- and over-nourished states. This is most likely due to the negative impact of SGLT1 deficiency on blood sugar. Conversely, disruption of GLUT4 does not lead to a decrease in blood glucose levels, thus anabolic hormones such as insulin remain elevated. We note that in our model energy loss is compensated by other nutrients, such as fatty acid oxidation, which is comparable to a resting state. During exercise, the effects of reduced glucose uptake in muscle would be more essential.

**FIGURE 6:**
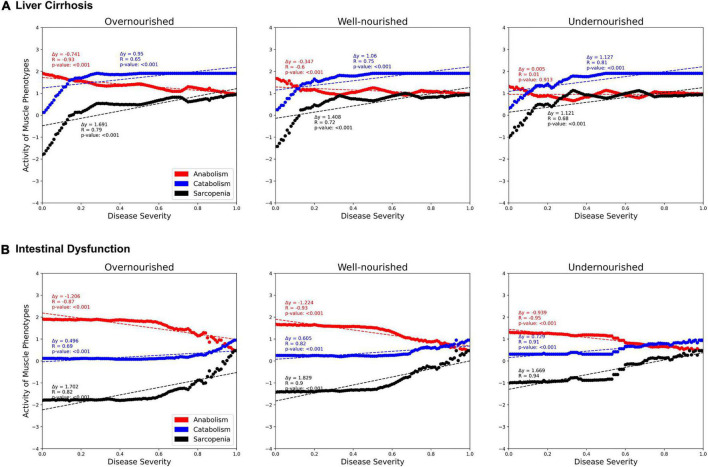
Predicted activities of three muscle phenotypes in response to increasing severity of liver cirrhosis (LC, **A**) and intestinal dysfunction (ID, **B**) in three different nutrition states. Each point represents a simulation in which, starting from an initial state, signal transduction is iterated over 100 consecutive steps. During these steps, the state of LC or ID is set active with a defined frequency representing their severity.

Next, we investigated the correlations between activities of LC and ID on the muscle phenotypes “anabolism,” “catabolism,” and “sarcopenia” dependent on the nutrition state (Figure 6). In both diseases, we see a strong positive correlation with catabolism (blue) and a negative correlation with anabolism (red). Thus, both disease states also correlate positively with sarcopenia. No major differences are observed between the nutrition states. However, the contribution of both diseases to anabolism appears to be lower in the malnourished state than in the other states. Presumably, this is due to the generally lower activity of anabolism in the undernourished state. In general, the correlation in the overnourished state tends to be constant, whereas the correlations in the nourished and undernourished states are more divergent. In these undernourished states, a greater increase in catabolic and sarcopenic activity is observed even at low LC activities (<0.2). Conversely, the sarcopenia phenotype in ID shows an almost plateau-like behavior at lower disease activities (<0.5), especially in malnourished states, and only then starts to increase. This is to be expected because at a low frequency of food intake, the baseline activity of sarcopenia is increased and the effects of ID, which is mainly related to food absorption, are minimal.

## Discussion

As scientific knowledge increases, so does awareness of the complexity of the molecular mechanisms that regulate biological processes. Gastrointestinal diseases are regulated through complex, interconnected networks in multiple cell types, tissues, and organs (49). Muscle growth and function are tightly regulated processes to keep the body functioning in different dietary situations (5, 14). Therefore, various nutrients and hormones are involved in regulating muscle activity, which complicates the search for the causes of dysregulations, such as sarcopenia. Moreover, the function of individual molecules in these processes can change depending on environmental conditions, such as the activity of other elements. Experimental setups are usually unable to simultaneously mimic the complex interactions between different metabolic pathways in various cells, compartments, or tissues. The complex molecular networks linking gastrointestinal diseases, MN, and sarcopenia motivate the use of *in silico* approaches.

We established the Sarcopenia Map to bring the complex molecular interaction pathways in sarcopenia into a comprehensive, standardized, and reproducible format. The Sarcopenia Map is a knowledge base that (i) gathers molecular information annotated with databases references, (ii) intuitively visualizes signal transductions, and (iii) provides tools for *in silico* simulations. By topologically evaluating the highly interconnected molecular network, users can utilize our tools to identify interaction paths between molecules of interest. Using Boolean simulations, the tool allows observing how changes in molecular activities propagate through the system and affect different compartments. However, it should be noted that Boolean models are divided into successive steps of discrete values and therefore cannot analyze continuous changes or molecular quantities. In our model, this limitation is less relevant because the correlation analysis observes changes in the system over multiple iterations rather than analyzing a single signal transduction. In this way, our model can identify and visualize mechanistic relationships across the entire network. Furthermore, we summarized subsequent intermediate reactions of pathways into a single element. This simplification allows us to keep a structured visualization and makes the model more robust toward feedback signaling. Retaining all reactions would distort the temporal perception of signal transduction. In a synchronously updated Boolean model such as ours, the time scales of all biological events are considered equally, thus more steps are required for pathways with more intermediate reactions. In reality, however, most reactions occur simultaneously because of the large quantities of molecules involved. For example, in *our muscle model only the ubiquitin-proteasome system is included as a junction of catabolic signals, as incorporating of all available regulatory processes would be too complex for the current focus of the map. Thus*, in developing our model, we aimed to strike a balance between feasibility and informativeness of the complex molecular interaction network. By successfully reproducing existing knowledge of the carbohydrate system in (patho-)physiological conditions, we showed that our model is capable of simulating such molecular processes.

We provide the community with a free-to-use platform to support nutrition research in developing or validating new hypotheses. While our work focused on the effects of gastrointestinal diseases, such as LC or ID in sarcopenia, the map itself provides a comprehensive knowledge base linking nutrition and muscle metabolism that can be also useful for other research areas. The hierarchical format of the map and the standardized representation of molecular interactions facilitate the extension to other related diseases or integration of new information, such as MN in relation to other tissues, in the future. Disease maps are community resources, and MINERVA provides tools for the community to expand these maps collaboratively. We encourage researchers to use the Sarcopenia Map to support open science by sharing scientific results and extending the map.

## Data availability statement

Publicly available datasets were analyzed in this study. This data can be found here: https://github.com/sbi-rostock/AIR/tree/master/SarcopeniaMap.

## Author contributions

OW, RJ, GL, and MW conceptualized and supervised the project. MH, LE, KB, and MW supervised parts that included literature research, curation of content, and layout of submaps. CS, DB, and VC performed literature research and designed the submaps. MH created the model, developed the tools, and performed the analyses. LE supervised both model design and interpretation of results, in the medical context. MH and LE prepared the initial version of the manuscript. OW, RJ, GL, KB, and MW critically evaluated the manuscript and the results. All authors contributed to the scientific content and helped to write the text and approved the submitted version.

## Footnotes

1
https://www.sbi.uni-rostock.de/sarcopenia

## Supplementary material

Supplementary Figure 1 SBML-standardized submap of muscle-specific processes involved in the regulation of sarcopenia. The map includes signaling pathways of hormones, cytokines, and metabolites on muscle anabolism **(left)** and catabolism **(right)**, thus regulating the development of sarcopenia (orange).

Supplementary Figure 2 SBML-standardized submap of liver-specific processes involved in the regulation of sarcopenia. The map contains information on metabolic processes **(left)**, secreted hormones or metabolites **(right)**, and their alterations in liver cirrhosis (orange).

Supplementary Figure 3 SBML-standardized submap of Gut-specific processes involved in the regulation of sarcopenia. The map contains information on nutrient resorption, secretion of hormones, and their alterations in intestinal dysfunction (orange).

Supplementary Figure 4 Steady state of the Boolean model with a constantly active “food intake.” Each dot represents an active element (*y*-axis) in the respective step (*x*-axis) during the steady state. In the last step the original state is reached, and thus the sequence iterates infinitely.

Supplementary Figure 5 Simulations of molecular perturbations and their observed correlation with other elements in the map. Each point represents a simulation experiment in which the respective nutritional state was simulated over 100 steps. During the simulation, the perturbed element was inactivated at a specific frequency (*x*-axis) and the activity of the observed element was measured. **(A)** Deficient glycogenolysis in the liver. **(B)** Deficient glucose uptake in the muscle through SLC2A4 (GLUT4). **(C)** Deficient glucose absorption in the intestine through SLC5A1 (SGLT1) without sucrose/fructose supplementation.

## Abbreviations

ID: Intestinal Dysfunction
LC: Liver Cirrhosis
MIM: Molecular Interaction Map
MN: Malnutrition
SBML: Systems Biology Markup Language
SBS: Short Bowel Syndrome
SP: Shortest Path.
