# Supplementary figures and images for: In silico investigation of molecular networks linking gastrointestinal diseases, malnutrition, and sarcopenia
Source: Front Nutr. 2022 Nov 4;9:989453. doi: 10.3389/fnut.2022.989453 (PMC9672470; doi:10.3389/fnut.2022.989453)

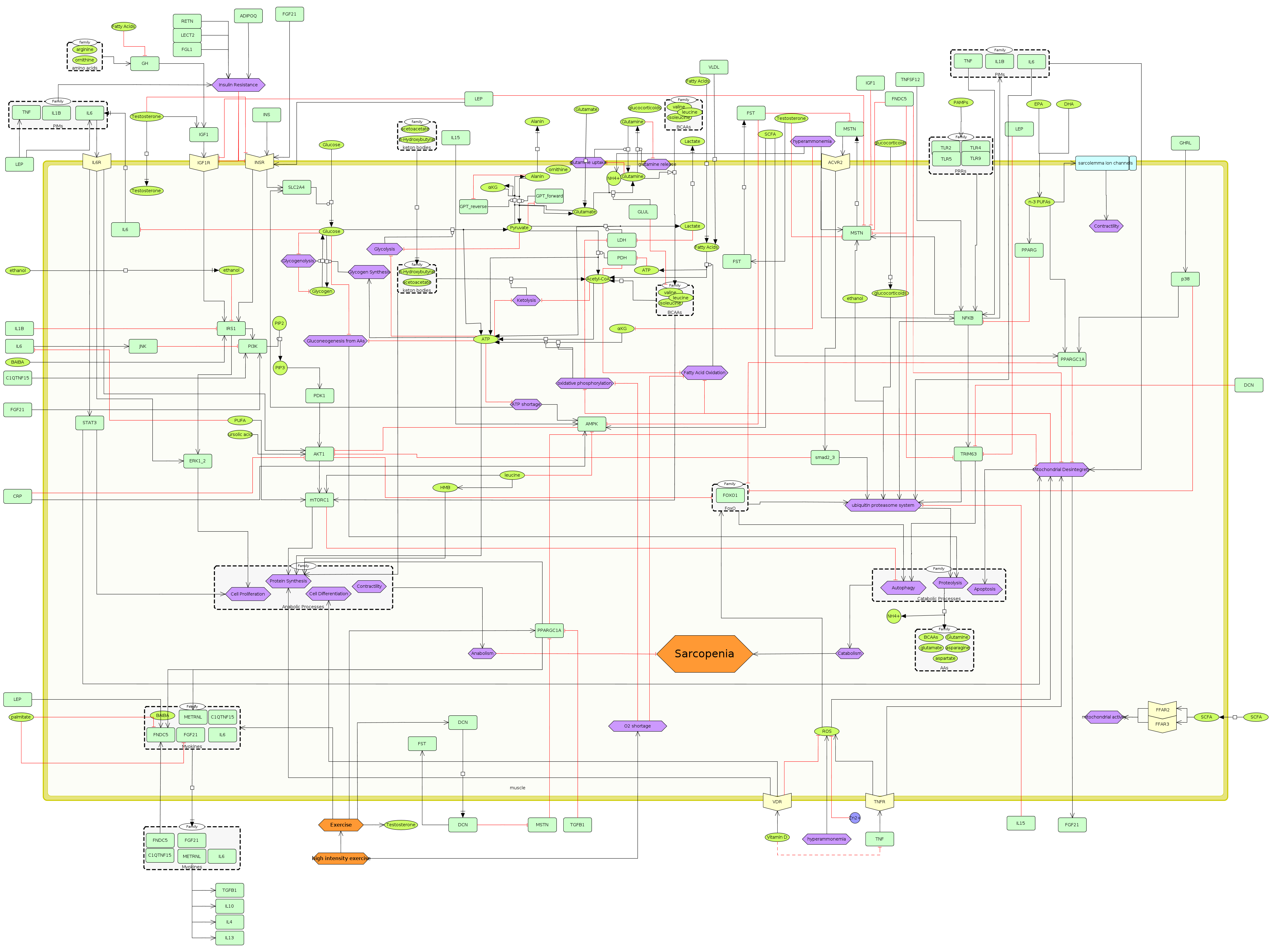

Supplement: Supplementary Figure 1 — SBML-standardized submap of muscle-specific processes involved in the regulation of sarcopenia. The map includes signaling pathways of hormones, cytokines, and metabolites on muscle anabolism (left) and catabolism (right), thus regulating the development of sarcopenia (orange). [file Image_1.JPEG]

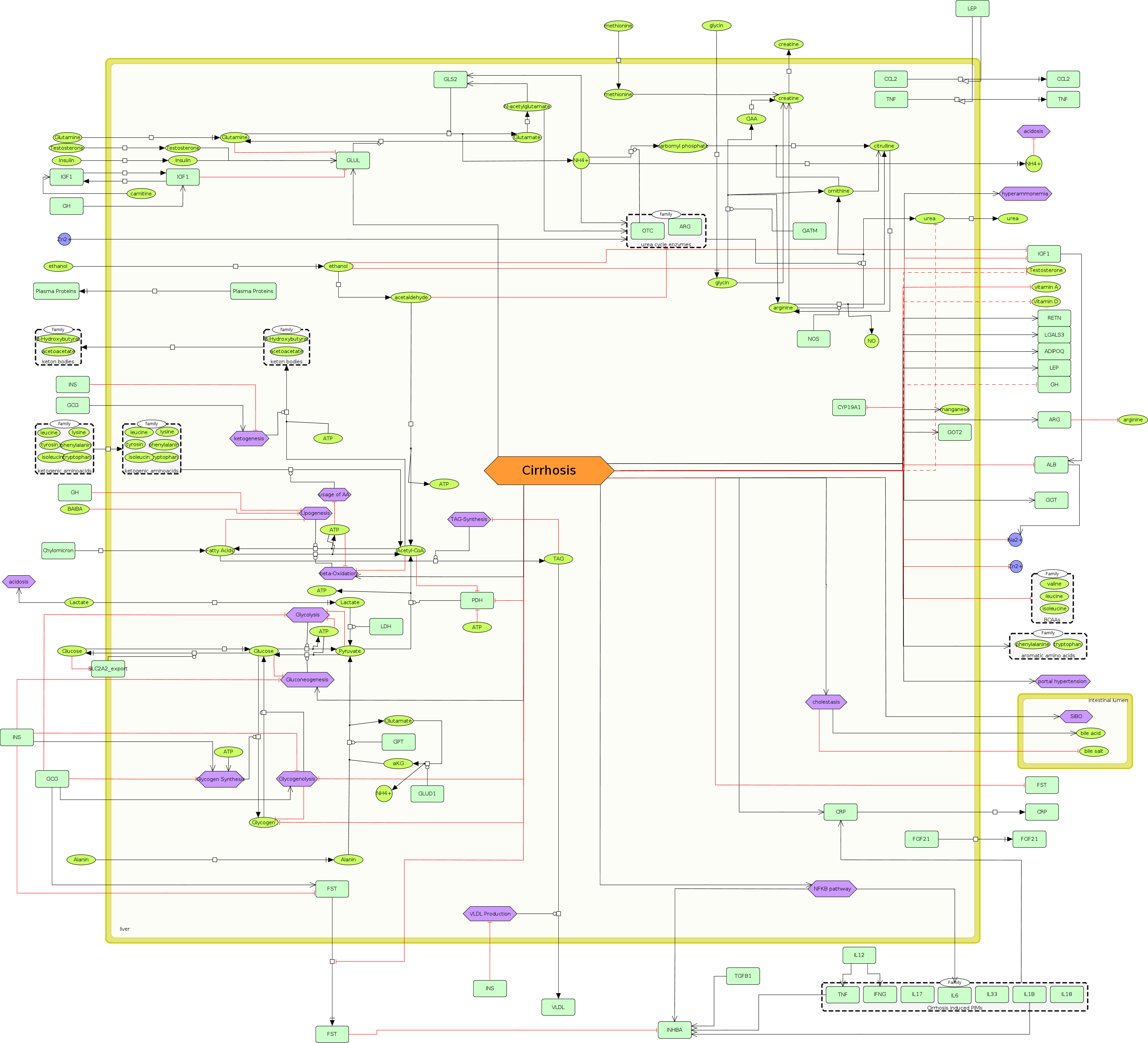

Supplement: Supplementary Figure 2 — SBML-standardized submap of liver-specific processes involved in the regulation of sarcopenia. The map contains information on metabolic processes (left), secreted hormones or metabolites (right), and their alterations in liver cirrhosis (orange). [file Image_2.JPEG]

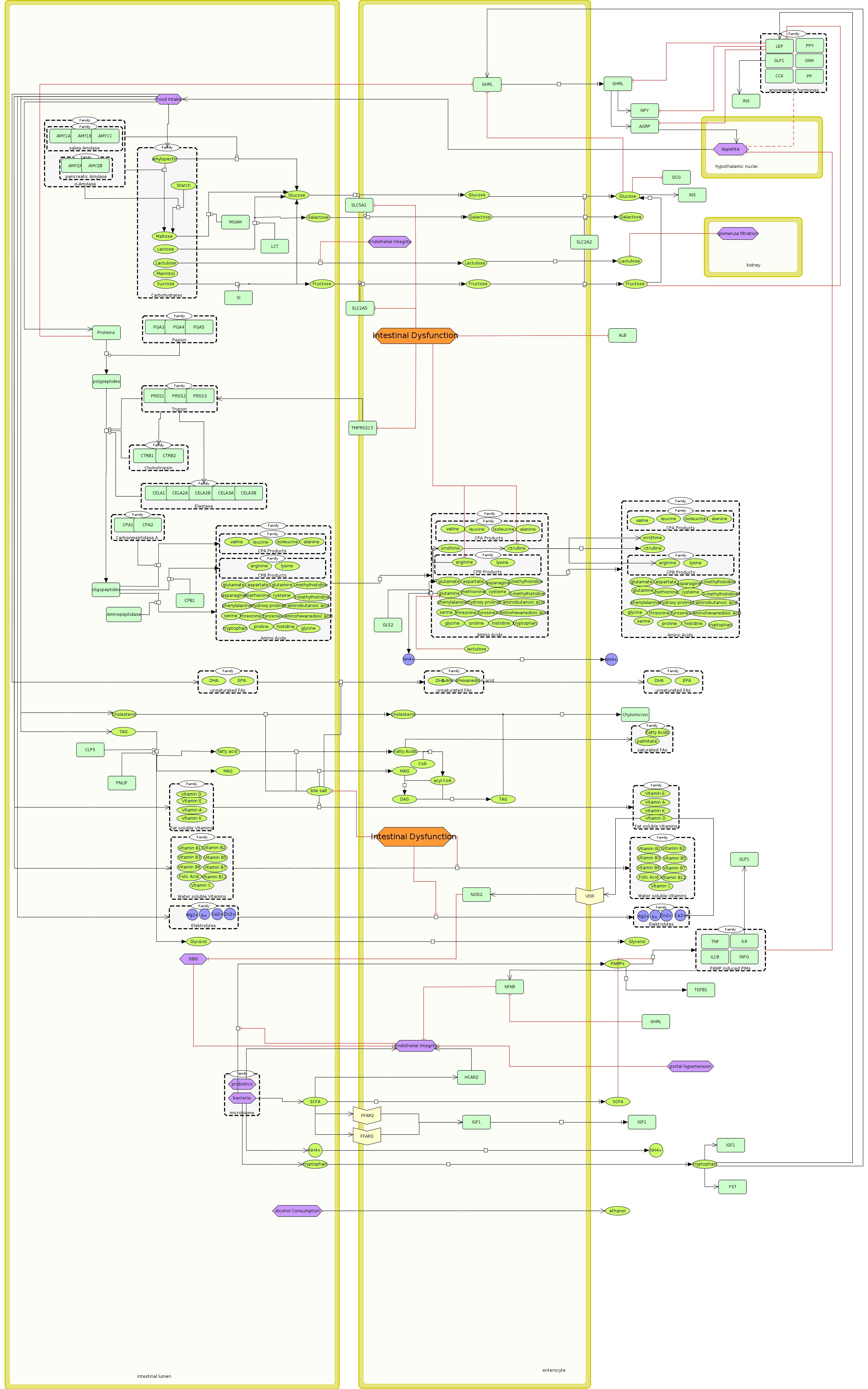

Supplement: Supplementary Figure 3 — SBML-standardized submap of Gut-specific processes involved in the regulation of sarcopenia. The map contains information on nutrient resorption, secretion of hormones, and their alterations in intestinal dysfunction (orange). [file Image_3.JPEG]

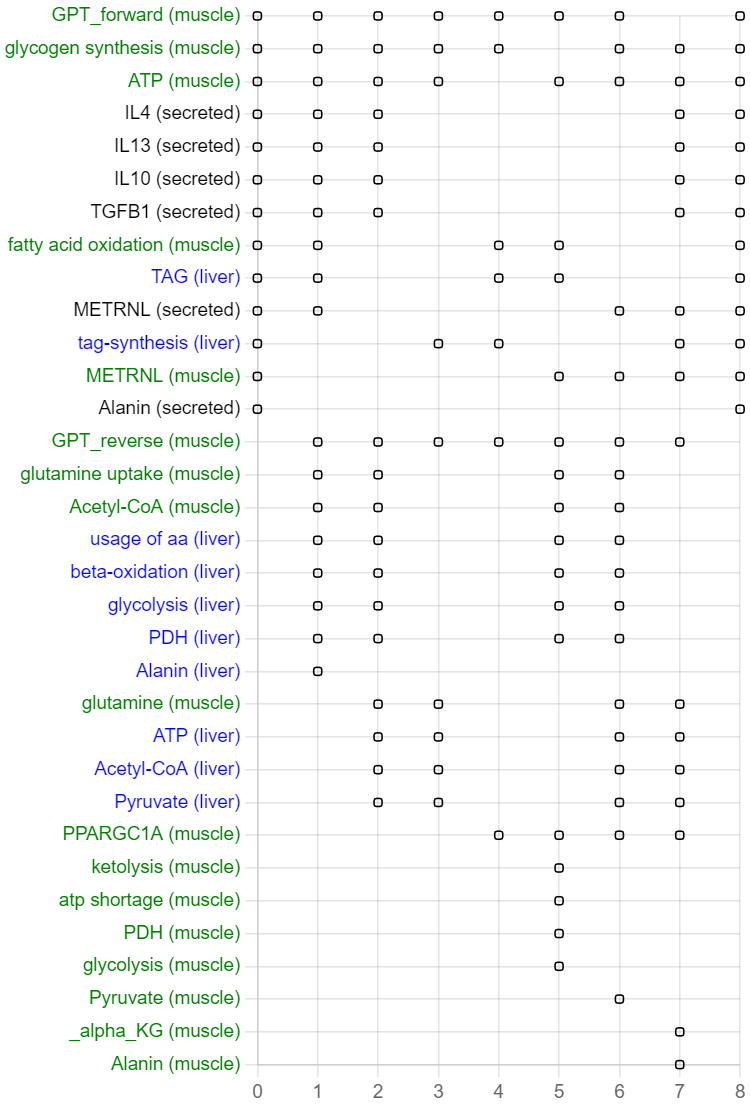

Supplement: Supplementary Figure 4 — Steady state of the Boolean model with a constantly active “food intake.” Each dot represents an active element (y-axis) in the respective step (x-axis) during the steady state. In the last step the original state is reached, and thus the sequence iterates infinitely. [file Image_4.JPEG]

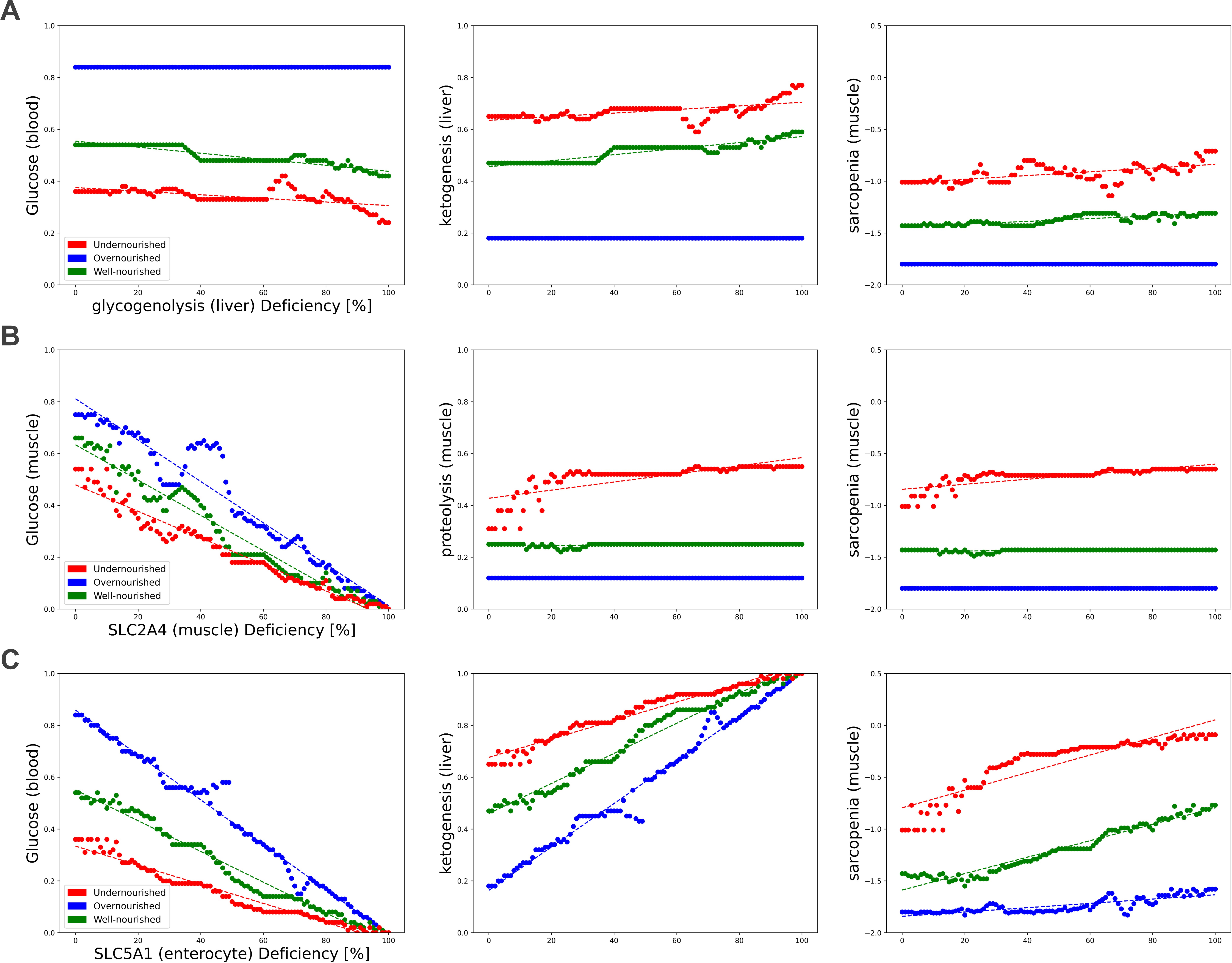

Supplement: Supplementary Figure 5 — Simulations of molecular perturbations and their observed correlation with other elements in the map. Each point represents a simulation experiment in which the respective nutritional state was simulated over 100 steps. During the simulation, the perturbed element was inactivated at a specific frequency (x-axis) and the activity of the observed element was measured. (A) Deficient glycogenolysis in the liver. (B) Deficient glucose uptake in the muscle through SLC2A4 (GLUT4). (C) Deficient glucose absorption in the intestine through SLC5A1 (SGLT1) without sucrose/fructose supplementation. [file Image_5.JPEG]
